# State Space Representation of Jiles–Atherton Hysteresis Model and Application for Closed-Loop Control

**DOI:** 10.3390/ma17153695

**Published:** 2024-07-26

**Authors:** Jiye Zhao, Jiqiang Zhou, Lu Zhang, Jinji Sun

**Affiliations:** 1 School of Instrumentation Science and Optoelectronics Engineering, Beihang University, Beijing 100191, China; jiyezhao@buaa.edu.cn; 2 Hangzhou Institute of National Extremely-Weak Magnetic Field Infrastructure, Hangzhou 310028, China; 3 Key Laboratory of Ultra-Weak Magnetic Field Measurement Technology, Ministry of Education, Beihang University, Beijing 100191, China; 13095906665@163.com; 4 Zhejiang Provincial Key Laboratory of Ultra-Weak Magnetic-Field Space and Applied Technology, Hangzhou Innovation Institute, Beihang University, Hangzhou 310051, China

**Keywords:** J-A model, hysteresis, state space representation, local linearization, closed-loop control

## Abstract

Hysteresis is a fundamental characteristic of magnetic materials. The Jiles–Atherton (J-A) hysteresis model, which is known for its few parameters and clear physical interpretations, has been widely employed in simulating hysteresis characteristics. To better analyze and compute hysteresis behavior, this study established a state space representation based on the primitive J-A model. First, based on the five fundamental equations of the J-A model, a state space representation was established through variable substitution and simplification. Furthermore, to address the singularity problem at zero crossings, local linearization was obtained through an approximation method based on the actual physical properties. Based on these, the state space model was implemented using the S-function. To validate the effectiveness of the state space model, the hysteresis loops were obtained through COMSOL finite element software and tested on a permalloy toroidal sample. The particle swarm optimization (PSO) method was used for parameter identification of the state space model, and the identification results show excellent agreement with the simulation and test results. Finally, a closed-loop control system was constructed based on the state space model, and trajectory tracking experiments were conducted. The results verify the feasibility of the state space representation of the J-A model, which holds significant practical implications in the development of magnetically shielded rooms, the suppression of magnetic interference in cold atom clocks, and various other applications.

## 1. Introduction

Magnetic materials find extensive applications in various fields such as precision manufacturing, aerospace, and medical devices [1,2,3]. Hysteresis is a fundamental phenomenon observed in magnetic materials and is characterized by the dependence of the magnetization state on the history of the applied magnetic field. Understanding and quantifying hysteresis behavior is crucial for various applications. In magnet design and electromagnetic device development, understanding and controlling hysteresis characteristics are crucial for enhancing performance [4]. In sensor technology, hysteresis phenomena are widely used in the production of magnetic sensors and magnetic storage devices [5]. In medical imaging, hysteresis effects are employed in magnetic resonance imaging (MRI) and other magnetic imaging techniques [6].

The hysteresis model is a mathematical model used to describe the hysteresis effect in magnetic materials. By modeling the hysteresis characteristics of materials, it is possible to better design and optimize magnetic systems and devices to meet various application requirements [7,8]. Hysteresis models typically use different mathematical equations to describe the relationship between magnetization intensity and an external field, mainly including models such as the Preisach model, Jiles–Atherton (J-A) model, Stoner–Wohlfarth model, and Prandtl–Ishlinskii (P-I) model. Among them, the Preisach model is a hysteresis model with memory effects and simulates the complex hysteresis characteristics of magnetic materials based on a series of nonlinear switching elements [9,10]. The J-A model, which is based on the dynamic behavior of microscale magnetic moments, simulates the magnetization process under the influence of an external field by describing the internal magnetic moments [11,12]. The Stoner–Wohlfarth model describes the magnetic moment reversal behavior of magnetic particles under an external field, assuming the particles have a single magnetic moment that only flips when the external field exceeds a specific threshold. This model provides a theoretical framework for explaining hysteresis loops and magnetic moment reversal in ferromagnetic materials [13]. The Prandtl–Ishlinskii model is based on combining multiple individual hysteresis elements through the composition of hysteresis operators to simulate the complex nonlinear behavior of materials or systems. The principle of this model lies in the effective capture of the nonlinear characteristics of materials or systems by appropriately adjusting and combining these hysteresis elements [14,15].

Among the numerous models developed to describe hysteresis phenomena, the J-A model is based on microscopic physical mechanisms, which makes its physical meaning clear and precise [16,17]. The J-A model incorporates parameters such as coercivity, remanence, and magnetic viscosity to characterize the hysteresis loop. Through empirical formulations and mathematical representations of energy dissipation mechanisms, the J-A model effectively captures the intricate interplay between magnetic domains, enabling accurate predictions of magnetization dynamics [18,19]. By calibrating the model parameters to experimental data, researchers can tailor the J-A model to specific material properties and geometries, enhancing its predictive capabilities across a wide range of applications [20,21]. Moreover, advancements in computational techniques facilitated the implementation of the J-A model in numerical simulations, allowing researchers to explore complex hysteresis phenomena in unprecedented detail [22,23].

To utilize the J-A model in practical applications, parameter identification is required. Currently, common methods for parameter identification in the J-A model can be categorized into two types: formula-based and fitting-based methods. Although the formula-based method has clear physical significance, it is highly sensitive to the selection of initial values and the order of parameter iteration. This sensitivity often leads to non-convergence or entrapment in local optima, resulting in low accuracy and efficiency in parameter identification [24,25]. The fitting-based method uses the least squares function of measured and calculated values as the objective function, and employs optimization algorithms to extract parameters of the J-A hysteresis model. Currently, various intelligent algorithms, such as the genetic algorithm (GA), simulated annealing, and particle swarm optimization (PSO), are applied to the parameter identification of the J-A model [26,27]. These algorithms offer advantages such as simplicity in implementation and high execution efficiency.

The numerical calculation of the J-A model is widely applied across various fields. For magnetostrictive materials, Rong et al. proposed a rational expression for the dynamic J-A model and introduced a numerical computation method to rapidly obtain high-precision model results [28]. Chen et al. proposed a modeling method for an axial flux permanent magnet hysteresis damper (APHD) based on the vector J-A model, which improves the calculation convergence and reduces the calculation time. A rapid identification method based on numerical techniques and genetic algorithms was employed to obtain the parameters of the J-A hysteresis model [29]. For magnetic particle imaging, Li et al. proposed a more accurate model to describe the dynamic magnetization of superparamagnetic iron oxide (SPIO) nanoparticles, which was termed the modified J-A model. This model was applied in x-space algorithms to enhance the image resolution, thereby improving the performance of magnetic particle imaging (MPI) in medical fields, including cardiovascular imaging [30,31].

In many applications, the J-A model also needs to be integrated into control systems. In the low-frequency external fields experienced by satellites in low Earth orbit, the placement of any magnetically sensitive devices within the magnetic shield should consider the hysteresis effects. Peng et al. introduced the J-A hysteresis model to predict and compute magnetization-induced magnetic fields and suppressed external interference by adjusting the current of compensating coils to maintain a stable field within the shield [32]. To achieve precise control of electromagnetic actuators, Rosenbaum et al. described hysteresis characteristics using both the J-A model and the Preisach model. They implemented feedforward control based on the inverse model and conducted experiments on a force-controlled electromagnet system [33]. Chen et al. combined the Jiles–Atherton model with a magneto-mechanical effect to develop a self-sensing model that effectively describes the relationship between magnetization and magnetostriction. The drive coil is used to detect the induced voltage caused by changes in magnetization. The integration of sensing and actuation functions results in a compact actuator structure capable of real-time actuation state sensing [34].

Hysteresis can impact the stability and accuracy of the system in many applications. Developing a dynamic model of magnetization and integrating it into the control system significantly aids in controller design and system simulation. Therefore, this study further established a state space representation of the J-A model based on the numerical calculation model and integrated it into a closed-loop control system to achieve trajectory tracking. First, based on the five fundamental equations of the J-A model, a state space representation was established through variable substitution and simplification. Further addressing the singularity problem at zero-crossing points, a locally linearized form was derived using an approximation method based on actual physical characteristics. The feasibility of the state space model was demonstrated through modeling and simulation using the S-function module of Simulink. Additionally, the PSO algorithm was employed for parameter identification based on the hysteresis loops obtained through COMSOL finite element software and tested on a permalloy toroidal sample, which confirmed the good approximation ability of the J-A state space model to actual hysteresis loops. Finally, a closed-loop control system was constructed based on the state space model, and trajectory tracking experiments were conducted. The state space representation of the J-A model holds significant practical significance for the design and analysis of systems containing hysteresis properties, such as transformer design, the active suppression of magnetic interference in cold atomic clock magnetic shielding systems, and the control of magnetostrictive materials.

## 2. State Space Representation of the J-A Model

### 2.1. J-A Hysteresis Model

The J-A model is a mathematical model that was designed to describe hysteresis phenomena and is applicable to the modeling and analysis of magnetic materials, such as soft magnetic materials and magnetic storage materials. In the fields of magnetic field control and magnetic device design, the J-A model is employed for predicting and analyzing hysteresis effects. The J-A model starts from the energy balance equation in the process of material magnetization and derives a set of differential equations that characterize the variation in magnetization intensity during the material magnetization process.

The J-A model [16,17] is based on the non-hysteretic magnetization curve. In this approach, it is possible to distinguish between irreversible and reversible domain wall displacements. Using the Langevin function, the description of non-hysteretic magnetization strength $M_{an}$ can be represented as

(1) $$M_{an}=M_{s}\left(\coth\left(\frac{H_{e}}{a}\right)-\frac{a}{H_{e}}\right)$$

where *a* represents the domain wall density in soft magnetic materials. $M_{s}$ stands for the saturation magnetization, which is the maximum magnetization that a material can achieve when subjected to an applied field. $H_{e}$ represents the effective field strength, which takes into account the interactions between magnetic domains. It can be expressed in terms of the field strength *H* and the total magnetization *M*

(2) $$H_{e}=H+\alpha M$$

where α represents the degree of field interaction between magnetic domains. The irreversible magnetization strength $M_{irr}$ corresponds to the defective domain portion within soft magnetic materials, which represents the irreversible component during the magnetization process. It can be calculated using the following equation

(3) $$\frac{dM_{irr}}{dH}=\frac{M_{an}-M_{irr}}{\delta k-\alpha \left(M_{an}-M_{irr}\right)}$$

where *k* represents the pinning factor, indicating the blocking effect or loss within magnetic domains. δ is the directional parameter, which takes a value of +1 when dH/dt>0 and −1 when dH/dt<0, with *t* representing time. The reversible magnetization strength $M_{rev}$ represents the reversible component generated by wall deformation at coupled sites under the influence of an external field. It can be expressed as follows:

(4) $$M_{rev}=c\left(M_{an}-M_{irr}\right)$$

where *c* represents the reversibility coefficient, $c\in \left[0,1\right]$. The total magnetization *M* can be expressed as

(5) $$M=M_{rev}+M_{irr}$$

### 2.2. State Space Representation

The above five equations constitute the hysteresis J-A model. Based on this, the state space representation is further established below. This contributes to the analysis and control of magnetic materials and hysteresis. State space equations are a mathematical model used to describe the behavior of dynamic systems. They are typically represented by a set of differential equations that describe how the system state changes over time. These equations detail the evolution of the system state and how the system responds to inputs to produce outputs. State space equations are widely employed in control system engineering, signal processing, and various engineering fields, and provide a comprehensive tool for the analysis and design of systems.

From Equations (1) and (2), the anhysteretic magnetization can be derived:

(6) $$M_{an}=M_{s}\left(\coth\left(\frac{H+\alpha M}{a}\right)-\frac{a}{H+\alpha M}\right)$$

This is an equation regarding $M_{an}$ with respect to *H* and *M*. From Equations (4) and (5), the following can be derived:

(7) $$M=cM_{an}+\left(1-c\right)M_{irr}$$

This is an equation representing *M* as a function of $M_{an}$ and $M_{irr}$. By observing that Equations (6) and (7) both involve three variables ($M_{an}$, $M_{irr}$, and *M*), it is evident that both $M_{an}$ and $M_{irr}$ can be expressed as functions of *M*. Using Equation (7), $M_{irr}$ can be expressed as a function of *M*, as follows:

(8) $$M_{irr}=\frac{1}{1-c}M-\frac{c}{1-c}M_{s}\left(\coth\left(\frac{H+\alpha M}{a}\right)-\frac{a}{H+\alpha M}\right)$$

Taking the derivative of the above equation with respect to *H*, the following can be obtained:

(9) $$\frac{dM_{irr}}{dH}=\frac{1}{1-c}\frac{dM}{dH}-\frac{c}{1-c}\frac{dM_{an}}{dH}$$

Combining Equations (3) and (9), the following can be obtained:

(10) $$\frac{1}{1-c}\frac{dM}{dH}-\frac{c}{1-c}\frac{dM_{an}}{dH}=\frac{M_{an}-M_{irr}}{\delta k-\alpha \left(M_{an}-M_{irr}\right)}$$

Moving dM/dH to the left side of the equation yields

(11) $$\frac{dM}{dH}=c\frac{dM_{an}}{dH}+\left(1-c\right)\frac{M_{an}-M}{\left(1-c\right)\delta k-\alpha M_{an}+\alpha M}$$

Furthermore, from

(12) $$\frac{dM_{an}}{dH}=\frac{dM_{an}}{dH_{e}}\frac{dH_{e}}{dH}=M_{s}\left(-\frac{1}{a}csch^{2}\left(\frac{H_{e}}{a}\right)+\frac{a}{H_{e}^{2}}\right)\left(1+\alpha \frac{dM}{dH}\right)$$

The derivation yields

(13) $$\frac{dM}{dH}=cM_{s}\left(-\frac{1}{a}csch^{2}\left(\frac{H_{e}}{a}\right)+\frac{a}{H_{e}^{2}}\right)\left(1+\alpha \frac{dM}{dH}\right)+\left(1-c\right)\frac{M_{an}-M}{\left(1-c\right)\delta k-\alpha M_{an}+\alpha M}$$

Moving dM/dH to the left side of the equation yields

(14) $$\frac{dM}{dH}=\frac{cM_{s}\left(-\frac{1}{a}csch^{2}\left(\frac{H_{e}}{a}\right)+\frac{a}{H_{e}^{2}}\right)+\left(1-c\right)\frac{M_{an}-M}{\left(1-c\right)\delta k-\alpha M_{an}+\alpha M}}{1-\alpha cM_{s}\left(-\frac{1}{a}csch^{2}\left(\frac{H_{e}}{a}\right)+\frac{a}{H_{e}^{2}}\right)}$$

It can be seen that the variables in Equation (14) include only $H_{e}$ and $M_{an}$, and both $H_{e}$ and $M_{an}$ are functions of *H* and *M*. Therefore, dM/dH can be entirely expressed as a function of *H* and *M*. Combining Equations (1), (2), and (14), the state space representation of the J-A model can be obtained. In time domain simulations, it can be used in the following form:

(15) $$\frac{dM}{dt}=\frac{cM_{s}\left(-\frac{1}{a}csch^{2}\left(\frac{H_{e}}{a}\right)+\frac{a}{H_{e}^{2}}\right)+\left(1-c\right)\frac{M_{an}-M}{\left(1-c\right)\delta k-\alpha M_{an}+\alpha M}}{1-\alpha cM_{s}\left(-\frac{1}{a}csch^{2}\left(\frac{H_{e}}{a}\right)+\frac{a}{H_{e}^{2}}\right)}\frac{dH}{dt}$$

**Figure 1:**
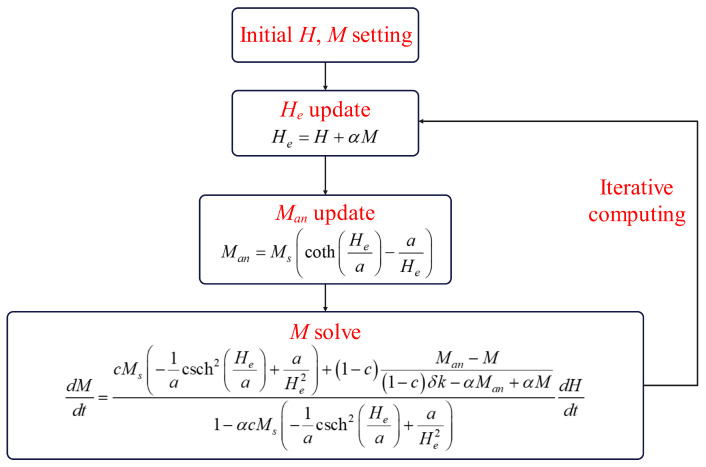
State space representation of the J-A model.

That is, $\dot{M}$ is a function of *M*, *H*, and $\dot{H}$: $\dot{M}=f\left(M,H\right)\dot{H}$. The state equation flowchart for the J-A model can be illustrated as shown in Figure 1.

With the powerful simulation capabilities of Simulink, this can be easily simulated. First, set the initial values for *H* and *M*. Next, calculate the value of $H_{e}$ and use the obtained $H_{e}$ to calculate the current $M_{an}$. Finally, iteratively calculate *M* using Equation (15), and use the new input *H*, along with *M*, as the initial input for the next iteration.

Consequently, the state space representation of the J-A model can be established. Define $u={\left[\begin{array}{cc} u_{1} & u_{1} \end{array}\right]}^{T}={\left[\begin{array}{cc} H & dH/dt \end{array}\right]}^{T}$ as the generalized input of the system, with the system state variable *x* and the system output *y* both being *M*. The state equation and output equation of the system can be expressed as follows:

(16) $$\left\{\begin{array}{c} \dot{x}=f\left(x,u_{1}\right)u_{2}\\ y=x \end{array}\right.$$

**Figure 2:**
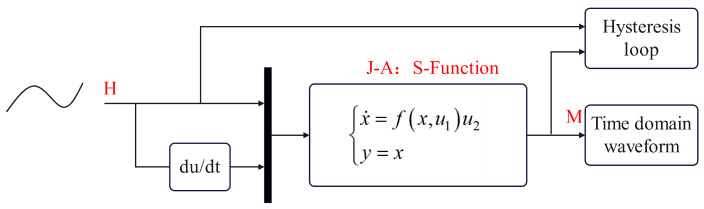
Simulation structure of the J-A model.

Using the S-function module in Simulink, the state equation can be modeled, as shown in Figure 2. The solution can be obtained by iteratively applying the Runge–Kutta method to reveal the material’s magnetization state.

The model outputs time domain waveforms. By taking *H* as the independent variable and *M* as the dependent variable, the hysteresis loop can be obtained. With the powerful features of Simulink and S-function, it becomes easy to modify the system inputs and parameters and perform the combined simulations with controllers, as well as stability testing. This contributes to both scientific research and engineering applications of hysteresis.

## 3. Local Linearization and Simulation

### 3.1. Local Linearization Based on L’Hopital’s Rule

The state space representation of the J-A model, derived as above, contributes to a better analysis and computation of hysteresis behavior. However, the presence of a singularity at $H_{e}=0$ in the expression of the J-A model can be inconvenient for its practical use. The actual initial magnetization curve of the material can be divided into the initial magnetization stage, the Rayleigh region, the maximum permeability region, the approaching saturation region, and the paramagnetic region. The initial magnetization stage represents the reversible magnetization stage under weak magnetic fields, where the magnetization intensity *M* maintains a linear relationship with the external field *H*. In this region, magnetic materials exhibit good reversibility, and the orientation of the magnetic moment can rapidly change without causing significant hysteresis losses. This is crucial for applications such as magnetic field control, magnetic storage, and magnetic sensing in magnetic materials. The width and characteristics of the reversible magnetization region typically depend on the magnetic properties of the material and the strength and direction of the external magnetic field. Therefore, based on the physical characteristics, the function value at $H_{e}=0$ is approximated and replaced. This is particularly meaningful for systems like a demagnetization system and a closed-loop control system, where achieving a convergence value of zero is crucial. Set

(17) $$f\left(H_{e}\right)=\coth\left(\frac{H_{e}}{a}\right)-\frac{a}{H_{e}}$$

Solve for the first and second derivatives of $f\left(H_{e}\right)$:

(18) $$f^{\prime }\left(H_{e}\right)=-\frac{1}{a}csch^{2}\left(\frac{H_{e}}{a}\right)+\frac{a}{H_{e}^{2}}$$

(19) $$f^{\prime \prime }\left(H_{e}\right)=\frac{2}{a^{2}}csch^{2}\left(\frac{H_{e}}{a}\right)\coth\left(\frac{H_{e}}{a}\right)-\frac{2a}{H_{e}^{3}}$$

Expand $f\left(H_{e}\right)$ using the Taylor series at the point $H_{e}=0$, as follows:

(20) $$f\left(H_{e}\right)=\frac{f^{\left(n\right)}\left(0\right)}{n!}{\left(H_{e}\right)}^{n}=f\left(0\right)+f^{\prime }\left(0\right)\cdot H_{e}+o\left(H_{e}\right)$$

The solution to this problem is challenging, and thus, we utilize L’Hopital’s rule for solving it. L’Hopital’s rule is a mathematical tool employed for calculating limits, and is particularly useful when encountering an indeterminate form like 0/0 in the process of finding limits. The basic idea of this rule involves taking the derivatives of both the numerator and denominator, then attempting to find the limit again, repeating the process until a meaningful result is obtained. This rule is particularly useful when dealing with some complex limit problems, especially in the computation of indeterminate fractional limits. First, by applying L’Hopital’s rule to approximate and solve for $f\left(0\right)$, set $x=H_{e}/a$, and thus,

(21) $$f\left(ax\right)=\coth\left(x\right)-\frac{1}{x}=\frac{x\left(e^{x}+e^{-x}\right)-\left(e^{x}-e^{-x}\right)}{x\left(e^{x}-e^{-x}\right)}$$

As $H_{e}$ approaches 0, the following can be obtained:

(22) $$\lim_{H_{e}\to 0}f\left(H_{e}\right)=\lim_{ax\to 0}f\left(ax\right)=\lim_{ax\to 0}\frac{x\left(e^{x}+e^{-x}\right)-\left(e^{x}-e^{-x}\right)}{x\left(e^{x}-e^{-x}\right)}$$

After two applications of L’Hopital’s rule, the following can be obtained:

(23) $$\lim_{ax\to 0}f\left(ax\right)=\frac{num^{\prime \prime }\left(x\right)}{den^{\prime \prime }\left(x\right)}=\frac{e^{x}-e^{-x}+x\left(e^{x}+e^{-x}\right)}{2\left(e^{x}+e^{-x}\right)+x\left(e^{x}-e^{-x}\right)}$$

where $num\left(x\right)$ represents the numerator of the original expression and $den\left(x\right)$ represents the denominator. From the results of the two applications of L’Hopital’s rule, it is evident that as *x* approaches 0, $num^{\prime \prime }\left(x\right)$ approaches 0, while $den^{\prime \prime }\left(x\right)$ approaches 4. Therefore, $\lim_{H_{e}\to 0}f\left(H_{e}\right)=0$. Next, by applying L’Hopital’s rule for local linearization to solve for $f^{\prime }\left(0\right)$, it follows that

(24) $$f^{\prime }\left(ax\right)=-\frac{1}{a}csch^{2}\left(x\right)+\frac{1}{a}\cdot \frac{1}{x^{2}}=\frac{1}{a}\cdot \frac{e^{2x}+e^{-2x}-2-4x^{2}}{x^{2}\left(e^{2x}+e^{-2x}-2\right)}$$

Performing four iterations of L’Hopital’s rule gives

(25) $$\lim_{ax\to 0}f^{\prime }\left(ax\right)=\frac{num^{\left(4\right)}\left(x\right)}{den^{\left(4\right)}\left(x\right)}=\frac{1}{a}\cdot \frac{8\left(e^{2x}+e^{-2x}\right)}{24\left(e^{2x}+e^{-2x}\right)+32x\left(e^{2x}-e^{-2x}\right)+o\left(x\right)}$$

From the results of the four iterations of L’Hopital’s rule, it can be determined that as *x* approaches 0, $num^{\left(4\right)}\left(x\right)$ approaches 8 and $den^{\left(4\right)}\left(x\right)$ approaches 24. Therefore, $\lim_{H_{e}\to 0}f^{\prime }\left(H_{e}\right)=1/3a$. Thus,

(26) $$f\left(H_{e}\right)=f\left(0\right)+f^{\prime }\left(0\right)\cdot H_{e}+o\left(H_{e}\right)\approx 0+1/3a\cdot H_{e}$$

Substituting $M_{s}/3a\cdot H_{e}$ for $M_{an}$, $M_{s}/3a$ for $dM_{an}/dH_{e}$, and H+αM for $H_{e}$ in Equation (11), the following can be obtained:

(27) $$\frac{dM}{dH}=c\frac{M_{s}}{3a}\left(1+\alpha \frac{dM}{dH}\right)+\left(1-c\right)\frac{H_{e}\frac{M_{s}}{3a}-M}{\left(1-c\right)\delta k-\alpha H_{e}\frac{M_{s}}{3a}+\alpha M}$$

Moving dM/dH to the left side of the equation yields

(28) $$\frac{dM}{dH}=\frac{cM_{s}\left[\left(1-c\right)\delta k-\alpha \left(H+\alpha M\right)\frac{M_{s}}{3a}+\alpha M\right]+\left(1-c\right)\left(H+\alpha M\right)M_{s}-\left(1-c\right)3aM}{\left(3a-\alpha cM_{s}\right)\left[\left(1-c\right)\delta k-\alpha \left(H+\alpha M\right)\frac{M_{s}}{3a}+\alpha M\right]}$$

It can be observed that as *M* and *H* approach 0,

(29) $$\lim_{M,H\to 0}\frac{dM}{dH}=\frac{cM_{s}}{3a-\alpha cM_{s}}$$

(30) $$\lim_{M,H\to 0}\frac{dM}{dt}=\frac{cM_{s}}{3a-\alpha cM_{s}}\frac{dH}{dt}$$

At this point, $\dot{M}=K\dot{H}$. This achieves the local linearization expansion at the zero-crossing point of the J-A model. This aligns with the objective physical phenomenon of material magnetization and provides a foundation for the analysis and design of systems containing hysteresis characteristics.

**Figure 3:**
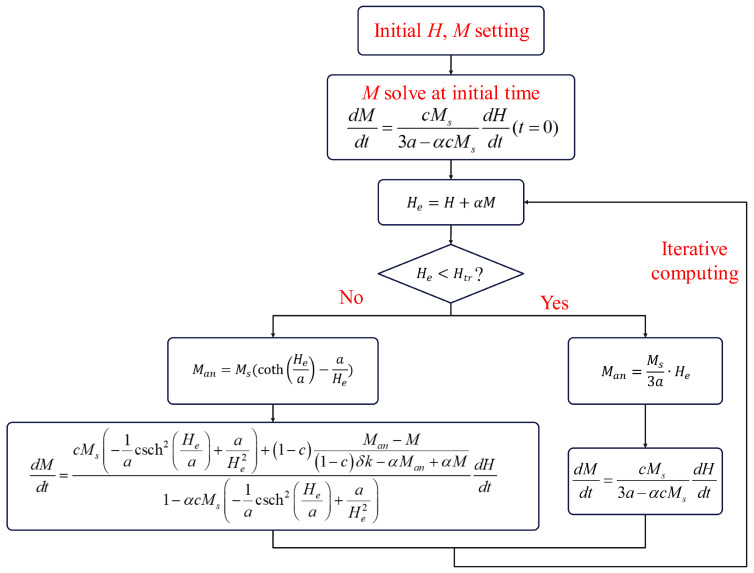
State space representation of the J-A model with local linearization.

To implement local linearization, it is necessary to preset a threshold $H_{tr}$, along with conditional statements. When the input value $H_{e}$ exceeds the threshold $H_{tr}$, calculations are performed according to the original formula; however, when the input value $H_{e}$ is less than or equal to the threshold $H_{tr}$, the calculation is carried out using the local linearization formula. Figure 3 illustrates the modified state flowchart incorporating local linearization.

**Figure 4:**
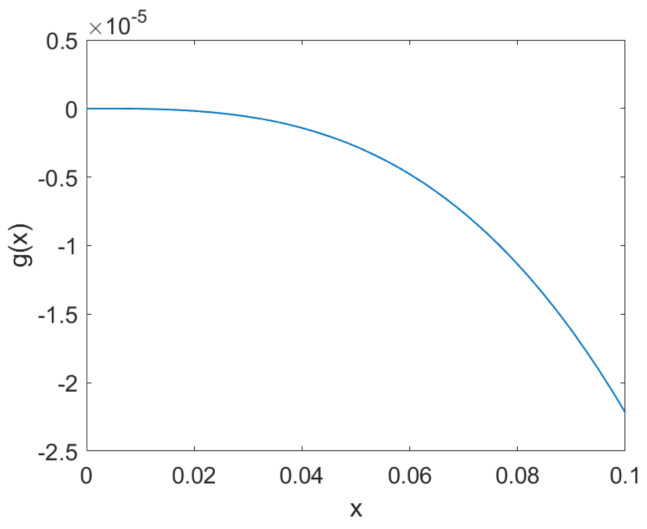
The error function between the Langevin function and the linear approximation.

Figure 4 shows the graph of the error function between the Langevin function and the linear approximation, i.e., g(x)=coth(x)−1/x−x/3. Since $x=H_{e}/a$, $H_{tr}$ can be chosen based on the iterative calculation step size and the linear approximation error.

### 3.2. S-Function Development and Simulation

Based on the analysis and derivation mentioned above, we further implemented the linearized J-A model state space representation, as follows:

(31) $$\left\{\begin{array}{c} \dot{x}=Ku_{2}\\ y=x \end{array}\right.$$

**Figure 5:**
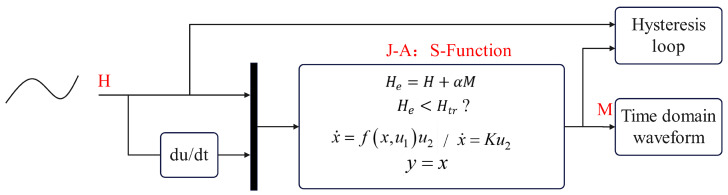
Simulation structure of the J-A model with local linearization.

The definitions of u, *x*, and *y* are the same as in Section 2.2. Define $u={\left[\begin{array}{cc} u_{1} & u_{2} \end{array}\right]}^{T}={\left[\begin{array}{cc} H & dH/dt \end{array}\right]}^{T}$ as the generalized input of the system, with the system state variable *x* and the system output *y* both being *M*. The simulation structure of the J-A model with the introduction of small-range linearization is shown in Figure 5. The only necessary modification involves supplementing the S-function module. The implementation of this only requires adding conditional statements, making it very easy to achieve. Therefore, a simulation model is established, and the iterative solution is obtained using the Runge–Kutta method.

**Figure 6:**
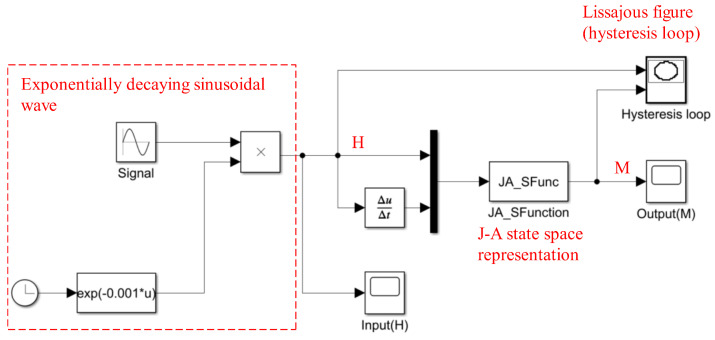
Simulink model with J-A S-function.

**Figure 7:**
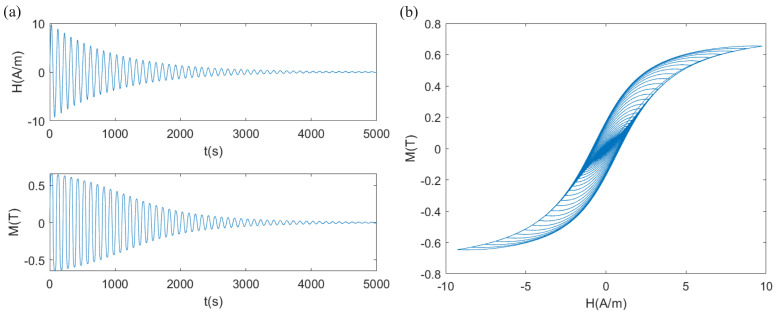
Calculation results of exponential decaying sinusoidal wave input: (**a**) time domain; (**b**) hysteresis loop.

By applying an exponentially decaying sinusoidal wave as the input, the Simulink model and results are shown in Figure 6 and Figure 7, respectively. The parameter settings are shown in Table 1.

As the amplitude of the input *H* decreased, the amplitude of the output *M* also decreased, gradually approaching zero. By plotting the Lissajous figure with *H* as the independent variable and *M* as the dependent variable, the hysteresis loop of the material could be obtained. The characteristics reflected by the curve matched the given parameter values. It can be observed that the hysteresis characteristics were well-described, and the singularity problem at the zero point was resolved. With this, the state space modeling of the J-A model was completed.

## 4. Hysteresis Loop Simulation and Parameter Identification of J-A State Space Model

### 4.1. Parameter Identification

Section 2 and Section 3 realized the state space representation and local linearization at the zero crossing point of the J-A model. To simulate the hysteresis behavior of practical magnetic materials, further identification of the five parameters in the J-A state space model is required. In this section, the hysteresis loops were constructed using COMSOL finite element software. Based on the hysteresis loops, parameter identification was carried out using the PSO method. The simulation model referred to the COMSOL official example library for modeling and simulation. The built-in J-A model was used to simulate and obtain the hysteresis loop with parameters identical to those in Table 1.

PSO is an evolutionary computation technique used for solving optimization problems. It simulates the behavior of swarms in nature, such as birds or fish, by continuously adjusting the positions and velocities of individuals to find the optimal solution. In PSO, each individual is called a particle, where its position represents a candidate solution in the solution space, and its velocity represents the direction and speed of the particle’s movement in the solution space. The core idea of PSO is to continuously update the positions and velocities of particles so that they move along the optimal direction in the search space and gradually converge to the global or local optimal solution. During this process, the movement of particles is influenced by their own historical best positions and the historical best position of the swarm, while also considering an inertia term to maintain the search diversity and convergence speed. The velocity and position update formulas for particles in the PSO algorithm are as follows:

(32) $$x_{d}^{k+1}=x_{d}^{k}+v_{d}^{k+1}$$

(33) $$v_{d}^{k+1}=\omega v_{d}^{k}+c_{1}r_{1}\left(p_{d}^{k}-x_{d}^{k}\right)+c_{2}r_{2}\left(p_{g}^{k}-x_{d}^{k}\right)$$

where *x* represents the position of the particle, which corresponds to the parameters of the J-A state space model that need to be solved. *v* represents the velocity of the particle, that is, the change in the iteration of the parameter values. ω is the inertia weight. $r_{1}$ and $r_{2}$ are two random numbers in $\left[0,1\right]$. $c_{1}$ is the local learning factor. $c_{2}$ is the global learning factor. The position vector $p_{d}^{k}$ is the optimal position of particle d after self-updating *k* times, which is called *PBest*. $p_{g}^{k}$ is the position of the best particle appearing after *k* updates in the particle swarm, which is called *GBest*.

**Figure 8:**
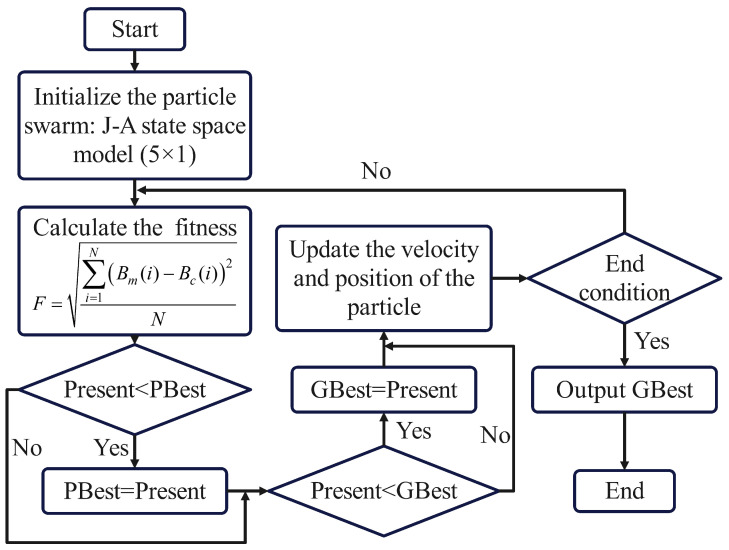
PSO algorithm flowchart.

The five parameters of the J-A state space model are taken as a multidimensional vector to be optimized. The fitness function is defined as the mean square error of the magnetic flux density at the sampling points, as follows:

(34) $$F=\sqrt{\frac{\sum_{i=1}^{N}{\left(B_{m}\left(i\right)-B_{c}\left(i\right)\right)}^{2}}{N}}$$

where $B_{m}$ represents the measured magnetic flux density value. $B_{c}$ denotes the computed magnetic flux density value obtained from the J-A state space model under the corresponding parameters. *N* is the total number of sampling points. *i* is the index of the sampling point. Based on these factors, the flowchart of the algorithm can be illustrated as shown in Figure 8.

### 4.2. Identification Result

The parameter configurations are defined as follows: the initial population consisted of 200 individuals, with a maximum iteration count of 300, ω=0.8, and $c_{1}=c_{2}=0.5$. The position range for the J-A state space model parameters $\left[\begin{array}{ccccc} M_{s} & a & \alpha & k & c \end{array}\right]$ spanned $\left[\begin{array}{ccccc} 1\times 10^{5}∼10\times 10^{5} & 0∼10 & 0∼10\times 10^{-5} & 0∼10 & 0∼1 \end{array}\right]$, while the velocity range varied over $\pm 0.1\times \left[\begin{array}{ccccc} 1\times 10^{5}∼10\times 10^{5} & 0∼10 & 0∼10\times 10^{-5} & 0∼10 & 0∼1 \end{array}\right]$. Finally, the identification result is shown in Table 2.

**Figure 9:**
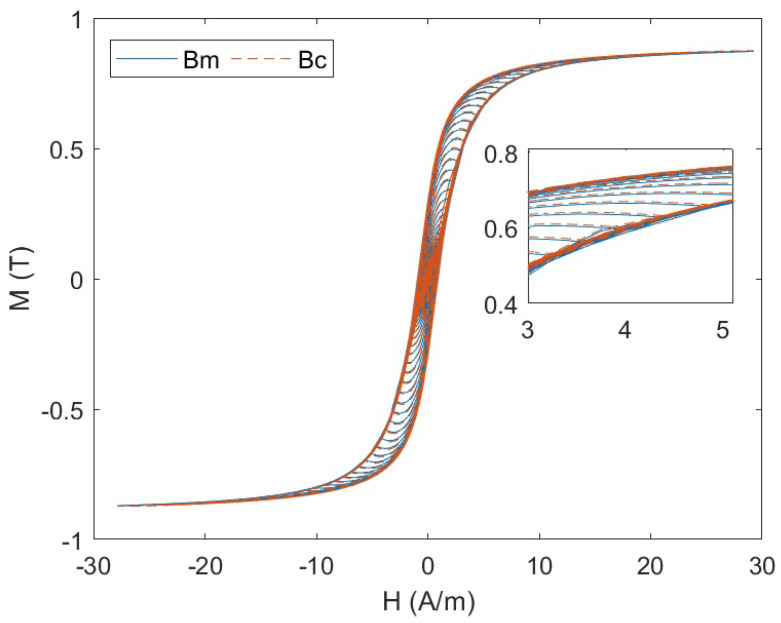
Fitting result of COMSOL-modeled data and J-A state space model.

As shown in Figure 9, the blue curve represents the hysteresis loop obtained from the COMSOL simulation result, while the red curve depicts the hysteresis loop generated using the parameters identified and the J-A state space model. It is evident that the two curves exhibit a good match, indicating a satisfactory fitting performance between them. This suggests that the combination of the J-A state space model proposed in this paper, along with the small neighborhood linearization method and the PSO parameter identification, can provide a satisfactory description of the hysteresis characteristics of magnetic materials.

**Figure 10:**
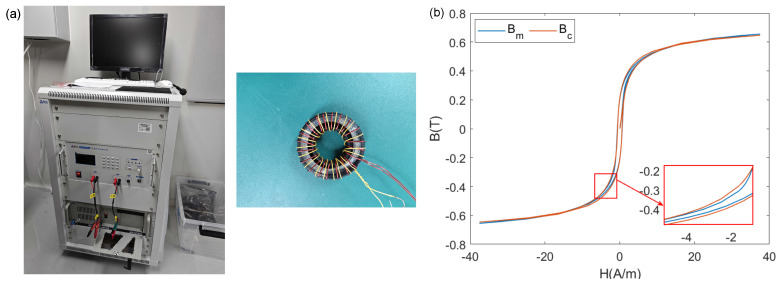
The testing device and fitting result of permalloy toroidal sample: (**a**) testing device; (**b**) fitting result.

To verify the fitting performance with the measured hysteresis loop, the permalloy toroidal samples were tested for their hysteresis loop using the MARS-3000S soft magnetic material DC measurement system from Hunan Linkjoin Technology Co., Ltd. (Loudi, China), as shown in Figure 10a. The testing method employed the simulated pulse technique. Excitation and induction coils were wound around the permalloy toroidal samples to apply the excitation magnetic field and generate the corresponding induced electromotive force. The identification result is shown in Table 3.

Figure 10b shows the fitting results of the parameters identified by the PSO. The blue curve indicates the measured hysteresis loop, while the red curve shows the fitting results of the J-A state space model. It can be seen that in most areas of the hysteresis loop, the fit was good, although there were some discrepancies in certain local regions. These errors existed whether using the J-A state space model or the classical iterative calculation method. In our opinion, this was due to the limitations of the primitive J-A model, sampling number of the testing device, or the PSO algorithm, and further targeted research is needed in the future. In a closed-loop control system, the impact of these errors can be effectively suppressed by the control algorithm.

The form of the state space representation and the application of local linearization hold significant practical implications in various fields. In the construction of magnetically shielded rooms, demagnetization of the high-permeability materials in the shielding layer is required, and the quality of the demagnetization directly affects the shielding performance. The proposed J-A state space model can effectively simulate the demagnetization process and integrate with the demagnetization system, aiding in the evaluation of the demagnetization performance. For applications such as cold atom clocks in the magnetic field experienced by satellites in low Earth orbit, magnetic hysteresis effects should be considered for magnetic-sensitive devices inside a shield. The proposed J-A state space model can effectively predict the field within the shield, thus achieving magnetic interference suppression combined with the coil system. In fields such as electromagnetic actuators, the proposed J-A state space model can also be used for system analysis and modeling, which provides a foundation for controller design.

## 5. Closed-Loop Control of J-A State Space Model

**Figure 11:**
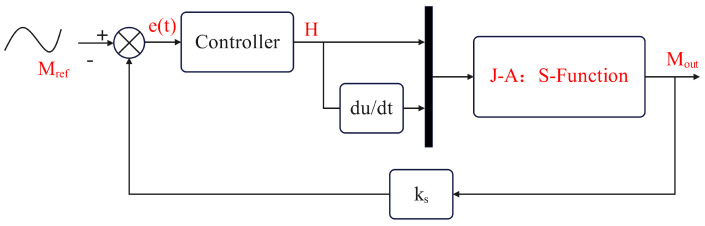
Closed-loop control system.

Through the above analysis and calculations, a state space representation of the J-A hysteresis model was established, and parameter identification was completed using the PSO method. This provides a foundation for the design of closed-loop control systems. Figure 11 represents the closed-loop block diagram of the system. The reference curve was fed into the closed-loop system and the difference with the feedback signal was taken to obtain the error signal e(t). $k_{s}$ is the feedback loop gain, which was set to 1. Through closed-loop control, this does not need to model the system precisely. Even in the presence of certain parameter deviations or external disturbances, the combination of feedback signals and the action of the controller allowed us to still output the desired set point.

**Figure 12:**
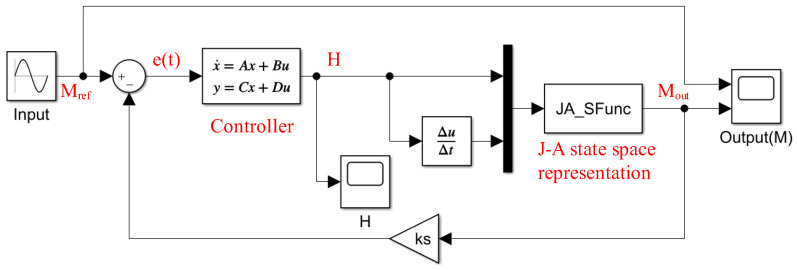
Simulink model of closed-loop control system.

The implementation of controllers, other components, and operations in the system can be easily achieved through modules in Simulink, as shown in Figure 12. The realization of the J-A model was based on the S-function implementation discussed earlier. This greatly facilitated the design and simulation of hysteresis control systems, which allowed for the validation of system characteristics under various input conditions and the presence or absence of external disturbances. It holds significant value for applications in engineering.

**Figure 13:**
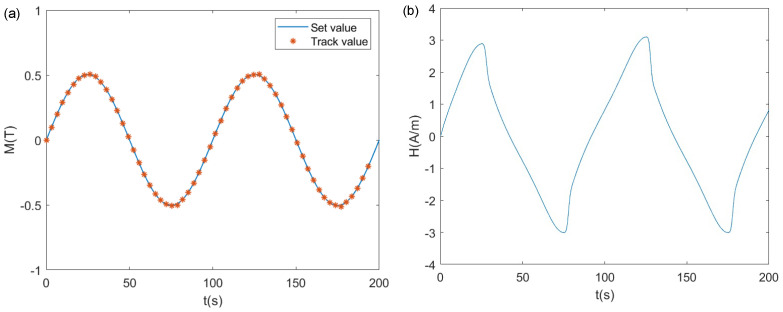
Tracking results of the sinusoidal wave input: (**a**) output variable *M*; (**b**) control variable *H*.

**Figure 14:**
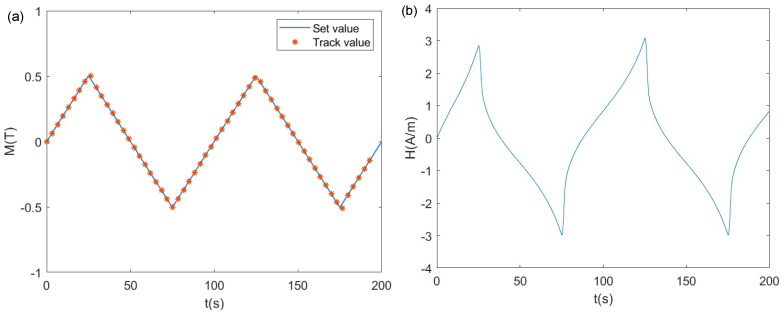
Tracking results of the triangular wave input: (**a**) output variable *M*; (**b**) control variable *H*.

The parameters for the J-A model were the same as in the third section. To achieve the regulation of hysteresis characteristics, the control was implemented based on the error e(t) and its integral ∫e(t)dt. The state variable *x* of the controller was defined as ∫e(t)dt, with the input *u* being e(t), and the output *y* formulated as $k_{p}e\left(t\right)+k_{i}\int e(t)dt$, as follows:

(35) $$\left\{\begin{array}{c} \dot{x}=u\\ y=k_{p}u+k_{i}x \end{array}\right.$$

where $k_{p}=2\times 10^{-6}$ and $k_{i}=5\times 10^{-4}$. A sinusoidal wave and triangular wave were used as system inputs for research. We selected the input signal in the Simulink source library and used the scope module to record the results. The results are shown in Figure 13 and Figure 14. In Figure 13a and Figure 14a, the blue curve represents the set value of the system, while the red asterisks denote the system output values. Figure 13b and Figure 14b depict the control variable of the system, namely, *H*. From the form of *H*, it can be seen that the control variable contained components of hysteresis nonlinearity.

It can be observed that the system output could effectively track the input. There was almost no deviation or lag between the system input and output. For the sinusoidal input tracking, the maximum error between the set value and the track value was only 0.0063 T, which was 1.26% of the sinusoidal input amplitude. This occurred near the peak of the sinusoidal wave, with the errors at other locations being less than 0.1%. When using a triangular wave, which had discontinuous points as the input, the maximum error between the set value and the tracking value was only 0.0091 T, which was 1.82% of the triangular wave input amplitude. Similarly, this occurred near the peak of the triangular wave, where the errors at other locations were less than 0.1%. The deviation and lag between the system output and input were both very small. This implies that the hysteresis state was successfully adjusted. It should be noted that PI control is not necessarily the optimal control method. Further research is needed to explore better design methods for the controller.

## 6. Conclusions

The J-A model is a commonly used model to describe the hysteresis characteristics of magnetic materials. This study established the state space representation based on the five basic equations of the J-A model. Furthermore, a small-range approximation linearization was applied to address the singularity issues using L’Hopital’s rule, which enhanced the integrity of the model. Subsequently, modeling was conducted using the S-function module in Simulink, and the feasibility of the model was verified using exponentially decaying sine waves and triangular waves as inputs. Then, parameter identification was performed using the PSO algorithm combined with COMSOL finite element software, which demonstrated that the proposed J-A state space model could effectively describe the hysteresis characteristic. Finally, a closed-loop control system was established. The tracking results for both the sinusoidal and triangular waves demonstrated that the system could effectively adjust the hysteresis state. This has significant practical implications in many areas, such as the development of magnetically shielded rooms and the suppression of magnetic interference in cold atomic clocks.

## Figures and Tables

**Table 1:** Parameters of J-A state space model.

Parameter	Value	Unit
$M_{s}$	$7.2\times 10^{5}$	A/m
*a*	1.15	A/m
α	$2.11\times 10^{-6}$	Dimensionless
*k*	2	A/m
*c*	0.47	Dimensionless

**Table 2:** The identification results of the simulation model.

Parameter	Value	Unit
$M_{s}$	$7.220\times 10^{5}$	A/m
*a*	1.146	A/m
α	$2.118\times 10^{-6}$	Dimensionless
*k*	1.977	A/m
*c*	0.466	Dimensionless

**Table 3:** The identification results of the measured data.

Parameter	Value	Unit
$M_{s}$	$5.70\times 10^{5}$	A/m
*a*	4.92	A/m
α	$2.42\times 10^{-6}$	Dimensionless
*k*	5.57	A/m
*c*	0.89	Dimensionless

## Data Availability

The raw data supporting the conclusions of this article will be made available by the authors on request.
